# Surface Drainage and Mulching Drip-Irrigated Tomatoes Reduces Soil Salinity and Improves Fruit Yield

**DOI:** 10.1371/journal.pone.0154799

**Published:** 2016-05-06

**Authors:** Maomao Hou, Lvdan Zhu, Qiu Jin

**Affiliations:** 1 College of Horticulture, Fujian Agriculture and Forestry University, Fuzhou, Fujian province, China; 2 Institute of Water Conservancy and Ecological Engineering, Nanchang Institute of Engineering, Nanchang, Jiangxi province, China; 3 Institute of Water Conservancy Science of Jiangsu Province, Nanjing, Jiangsu province, China; Purdue University, UNITED STATES

## Abstract

A study on the effects of mulched drip irrigation combined with surface drainage on saline soil and tomatoes was conducted in coastal areas of eastern China, where the crops are subjected to excessive salt. The treatments contained three irrigation rates—200, 250 and 300 m^3^/ha—and three drain ditch depths—10, 20 and 30 cm. The contents of soil salinity, organic matter and available nutrient were observed, and the tomato plant height, stem diameter and leaf area index during different growth periods were recorded. Results showed that the total removal rate of salt from soil at a 0–1 m depth was 8.7–13.2% for the three drainages. Compared with the control, the treatments increased the content of available N (by 12.1–47.1%) and available K (by 5.0–21.9%) in the soils inside the mulch and decreased the content of available N (by 3.4–22.1%) and available K (by 7.5–16.4%) in the soils outside the mulch. For tomatoes, the plant height and the stem diameter was increased significantly by the irrigations but was not significantly affected by the drainages, and the leaf area index was increased by 0.39~1.76, 1.10~2.90 and 2.80~6.86 respectively in corresponding to the seedling, flowering and fruit-set stage. Moreover, yield-increase rates of 7.9–27.6% were found for the treatments compared to the control with a similar amount of applied water.

## Introduction

In the coastal area of eastern China, the proportion of agricultural land, particularly cultivated land, is small, and it continues to decrease due to urban expansion [1–2]. To alleviate the shortage of cultivated lands, the exploitation and utilization of coastal shoal resources has received increasing attention. Hangzhou Bay District, which is located in Ningbo City in the Zhejiang Province of China, has abundant costal saline lands. The soil of Hangzhou Bay is deep and well-distributed (according to the bulk density). The average salinity of the 0–100 cm soil is 1–4‰, and in some areas, it reaches a relatively high value of 20–30‰ [3]. Although the soil conditions in Hangzhou Bay have improved considerably through an extended period of crop planting and freshwater washing [4], the excessive soil salts, particularly those in the plough layer, still require further processing.

To improve the condition of salt-affected soil, many methods have been proposed, including soil replacement [5], subsurface drainage [6], straw mulch [7–8], and bio-organic fertilizer [9]. Mulched drip irrigation cannot radically remove salt from soil, but it effectively suppresses the upward movement of salt to the plough layer. A field experiment on mulched drip irrigation showed that the soil salinity, the contents of individual salt ions, the pH value, the ratio of Cl^-^ to SO_4_^2-^ and the sodium adsorption ratio (SAR) in the 0-40-cm layer were significantly decreased by controlling the soil matric potential at a 20-cm depth [10]. Long-period mulched drip irrigation has been shown to decrease the soil bulk density (from 1.71 g/cm^3^ to 1.44 g/cm^3^) and to increase the saturated soil water content (from 20.3% to 30.2%) of the 0-10-cm layer correspondingly [11]. Moreover, the absolute amount of all microbial groups was shown to increase under mulch cropping [12]. Although positive effects of mulched drip irrigation on salt-affected soil were obtained, large amounts of soil nutrients were reported to be lost when excessive irrigation occurred during the crop-growth period; this was especially the case for the crop-establishment period [13]. Surface drainage is also applied to improve salt-affected soils. Salts in 0-100-cm soil obviously decreased in response to the establishment of drain ditches to conduct surface drainage [14]. However, unconscionable surface drainage usually causes large quantities of soil N and P loss, which is harmful to the surrounding water environment [15–16].

Under conditions of high soil salinity, many crop plants, including tomatoes, are susceptible; they cannot survive, or they can survive only with decreased yields. Mulched drip irrigation has been widely used to alleviate the deleterious effects of salinity on tomato [17–18]. Mulched drip irrigation has various effects on the nutrient components of tomatoes, and it plays a more significant role in increasing fruit yield than flood irrigation [19]. The total root length was found to be longer under drip irrigation than under flood irrigation [20]. Moreover, the irrigation schedule cannot be ignored as it affects the yield formation including the number and water content of fruit [21].

Presently, many studies have investigated the performance of saline soil or crops individually under mulched drip irrigation and surface drainage. However, few studies have looked into the effects of mulched drip irrigation combined with surface drainage on soil and crops in salt-affected fields. In this study, we chose tomatoes, which are sensitive to the variation of soil salt, as the plant material. The tomatoes were treated with different rates of drip irrigation and different ditch depths of surface drainage. The objectives of this study were to analyse the soil’s salt, available nutrients and organic matter response and the tomatoes’ growth and yield response under the combined treatment of mulched drip irrigation and surface drainage. These were compared with a flood irrigation treatment that has no surface drainage.

## Materials and Method

### Experimental site

The experiments were conducted from June to September of 2015 in Hangzhou Bay (Ningbo, latitude 30°10′N, longitude 121°13′E), China (The experiment was permitted by the owner of the land named He Han). The experiment site enjoys a subtropical, mild climate with four distinctive seasons. The mean annual temperature of Ningbo from 1961–2010 was 16.4°C. The temperature is highest in July, with a mean annual value of 28°C, and lowest in January, with a mean annual value of 4.7°C. The frost-fee period is 230–240 days. The mean amount of annual sunshine hours is 1850 h. Moreover, the experimental site has a mean annual precipitation of 1480 mm, and most of the precipitation (60%) occurs between May and September. The experiment field was located at the Modern Agricultural Park of Development Zone of Hangzhou Bay. The soils (Alfisoils) in the field were mixed uniformly before the experiment. The soil type of the experimental fields was medium-textured soil with a bulk density of 1.46 g/cm^3^, organic matter of 0.928% at 0–60 cm, 3.47 g/kg of total salt at 0–60 cm, 44.33 mg/kg of available nitrogen, 52.10 mg/kg of available phosphorus, and 151.81 mg/kg of available potassium.

### Plant material and arrangement

**Fig 1:**
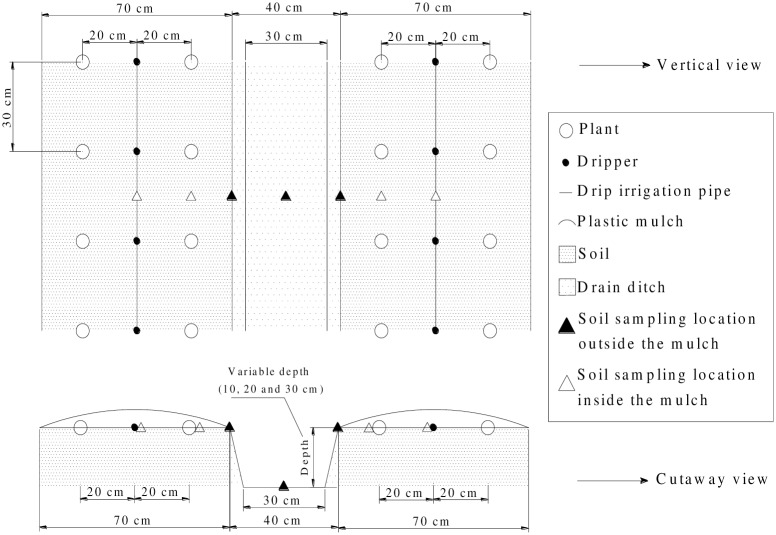
The arrangement of plants and the locations of soil samples.

The tomato variety “*Red Crown*” that purchased commercially from Nanjing Institute of Vegetable Science, Jiangsu province, China, was chosen. Seedlings were transplanted into the fields on June 10. These seedlings were arranged with 30-cm plant spacing and 40-cm row spacing. Two lines of tomatoes were irrigated by one drip pipe between them, and they were mulched with a single white, plastic film. The arrangement of the tomato plants is displayed in Fig 1.

Before being transplanted, the tomatoes were fertilized with 650 kg/ha compound fertilizer (N: P_2_O_5_: K_2_O = 1:2:2). Other field-management procedures were applied equally to all tomatoes. No additional light, heat, or CO_2_ were provided.

During the growth stage of the tomatoes, the lateral branches were removed timely. Each tomato plant was allowed to reserve 4 fruit sequences. Pest control was conducted aperiodically based on the actual conditions occurring in the experimental fields.

### Treatments and experimental design

Ten treatments were used to evaluate the combined effects of mulched drip irrigation and surface drainage on the saline soils and tomatoes. Each treatment occupied an area of (1.8×6) m^2^, and total area of the experimental field was 520 m^2^. Different treatments were arranged in one line. These treatments were distinguished by different rates of drip irrigation and different depths of drain ditches. The irrigation quotas were kept at three levels: 200 (I1), 250 (I2) and 300 m^3^/ha (I3). Pressure compensating emitters (produced by Runtian Water-saving Irrigation Equipment Co.,Ltd.) were adopted, with a distance of 30 cm between two emitters in one pipe. The drip flow was 2.4 L/h, and the amount of irrigation water was controlled by the irrigation duration. The drain ditches were excavated as the same top width of 40 cm and bottom width of 30 cm and as three different depths of 10 (D1), 20 (D2) and 30 cm (D3). A flood-irrigation treatment with a 250 m^3^/ha quota but no surface drainage was adopted as the control (CK). Each treatment was replicated three times. The treatments are shown in Table 1.

**Table 1:** Experimental design.

Treatment	I1D1	I2D1	I3D1	I1D2	I2D2	I3D2	I1D3	I2D3	I3D3	CK
Irrigation quota (m^3^/ha)	200	250	300	200	250	300	200	250	300	250
Depth of drain ditch (cm)	10	10	10	20	20	20	30	30	30	0

The tomatoes were irrigated every 7 days starting 2 DAT (Days after transplanted), as was shown in Table 2. Throughout the entire growth stage, the tomatoes were irrigated thirteen times. The surface drainages were conducted three times through ditches—35, 63 and 102 DAT (Table 2)—with a drainage flow of 3.2 L/s. The drainage duration was 30 min each time. On 35 and 63 DAT, the surface drainage was directly conducted; on 102 DAT, it was carried out after uncovering the mulch. Otherwise, simple rainproof facilities were installed in the experiment fields to prevent the precipitation from influencing the soil moisture during the experiment.

**Table 2:** The date of irrigation, drainage and soil sampling.

DAT	2	9	16	23	30	31	35	37	44	51
Irrigation	√	√	√	√	√			√	√	√
Drainage							√			
Soil sampling					√	√				
DAT	58	59	63	65	72	79	86	87	102	103
Irrigation	√			√	√	√	√			
Drainage			√						√	
Soil sampling	√	√					√	√	√	√

Note: DAT represented days after transplanted, Seedlings were transplanted into the fields on June 10.

### Samples and measurements

Each treatment had seven representative sampling points. These points were in a single line perpendicular to the drip pipe. The sampling points were located separately at the midpoint between the two drippers, the midpoint between the two tomato plants, the outer edge of the mulch and the middle of the drain ditch. Furthermore, the sampling points were divided into two different categories: the sampling locations outside the mulch (SOM) and the sampling locations inside the mulch (SIM), as marked in Fig 1. The soils at 0-60-cm depth were collected using a soil auger; then, these soils were homogenized and air-dried to measure the soil salinity. Similarly, the soils at a 0-20-cm depth were collected and treated to measure the content of available N, available P, available K and organic matter in soil [22–23]. The sampling dates of the soil used for the salt analysis in the following text were 30 and 31 DAT, 58 and 59 DAT, 86 and 87 DAT, 102 and 103 DAT, and the soil used for the organic matter and available nutrient analysis was sampled on 103 DAT.

Among the four lines of tomatoes in one treatment, the two lines closest to the drain ditch were used to observe the tomato yield. At each harvest time, the tomatoes’ number and weight were recorded, and the tomato yield was calculated after the last harvest. At 10, 30 and 60 DAT, six representative tomato plants were randomly chosen from one treatment to measure the plant height, stem diameter and leaf area index.

### Statistical analysis

The data were compared statistically in SPSS software Version 17.0 [24].

## Results

### Soil salinity

**Fig 2:**
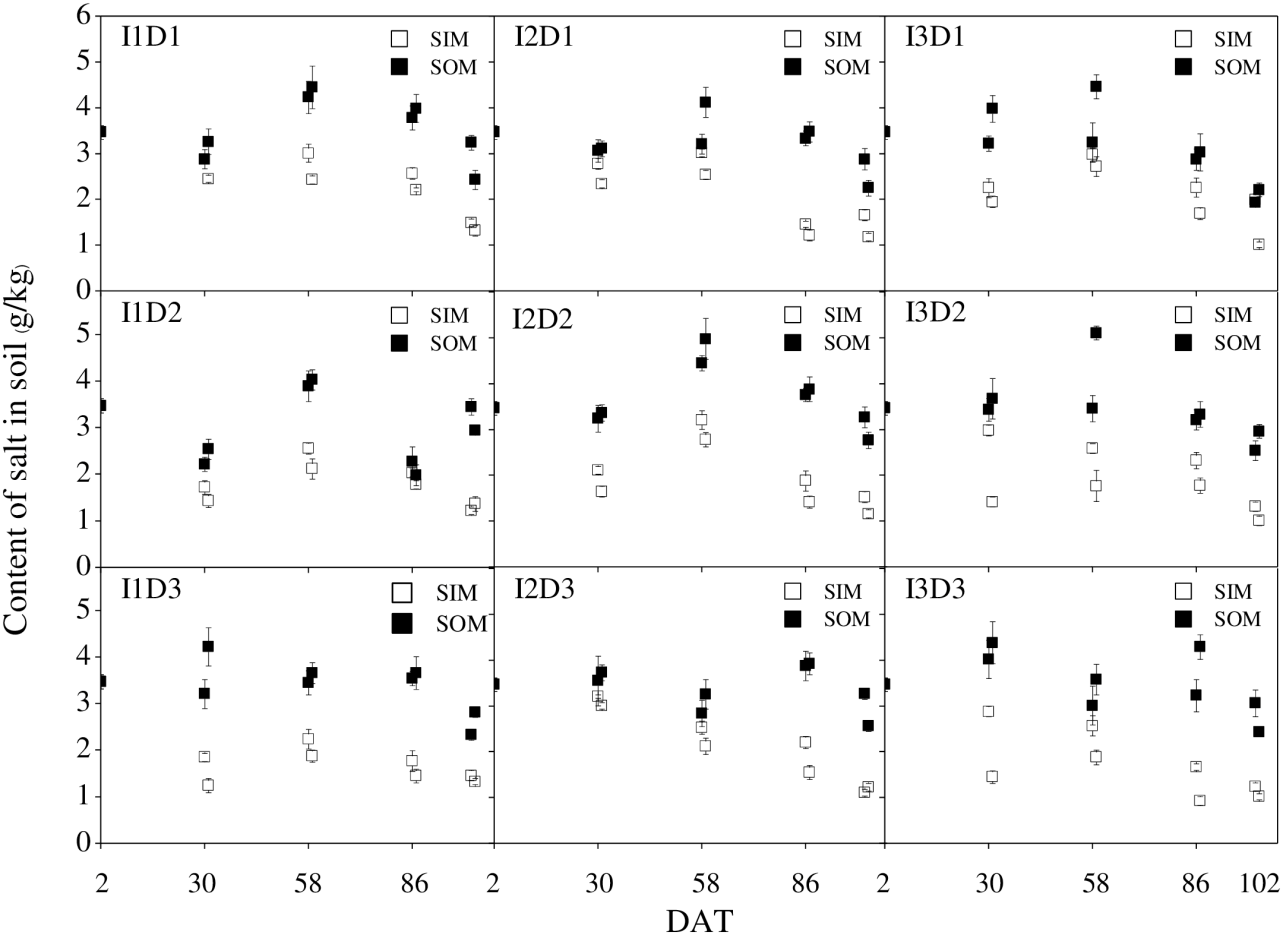
Variations of soil salinity with different treatments. (SIM and SOM represent the sampling location inside and outside the mulch, respectively.). DAT represented days after transplanted, Seedlings were transplanted into the fields on June 10.

The variations in the soil salinity are shown in Fig 2 for the nine treatments, as measured in the experiment. Regularities in the variation of the soil salt were similar across the different treatments, for both SIM and SOM. During the experiment, the soil salinity in SIM was obviously lower than that in SOM. After irrigation, the salt content of soil in SIM was decreased, whereas that in SOM was increased; this variation of salt content under I3 appeared to be more dramatic than that under I1 and I2. However, the variation tendency of the salt in both SIM and SOM was not apparent after the surface drainage. On 103 DAT, the soil salinity in SIM was decreased to 1.01–1.37 g/kg with different treatments, and that in SOM was decreased to 2.21–2.96 g/kg.

**Fig 3:**
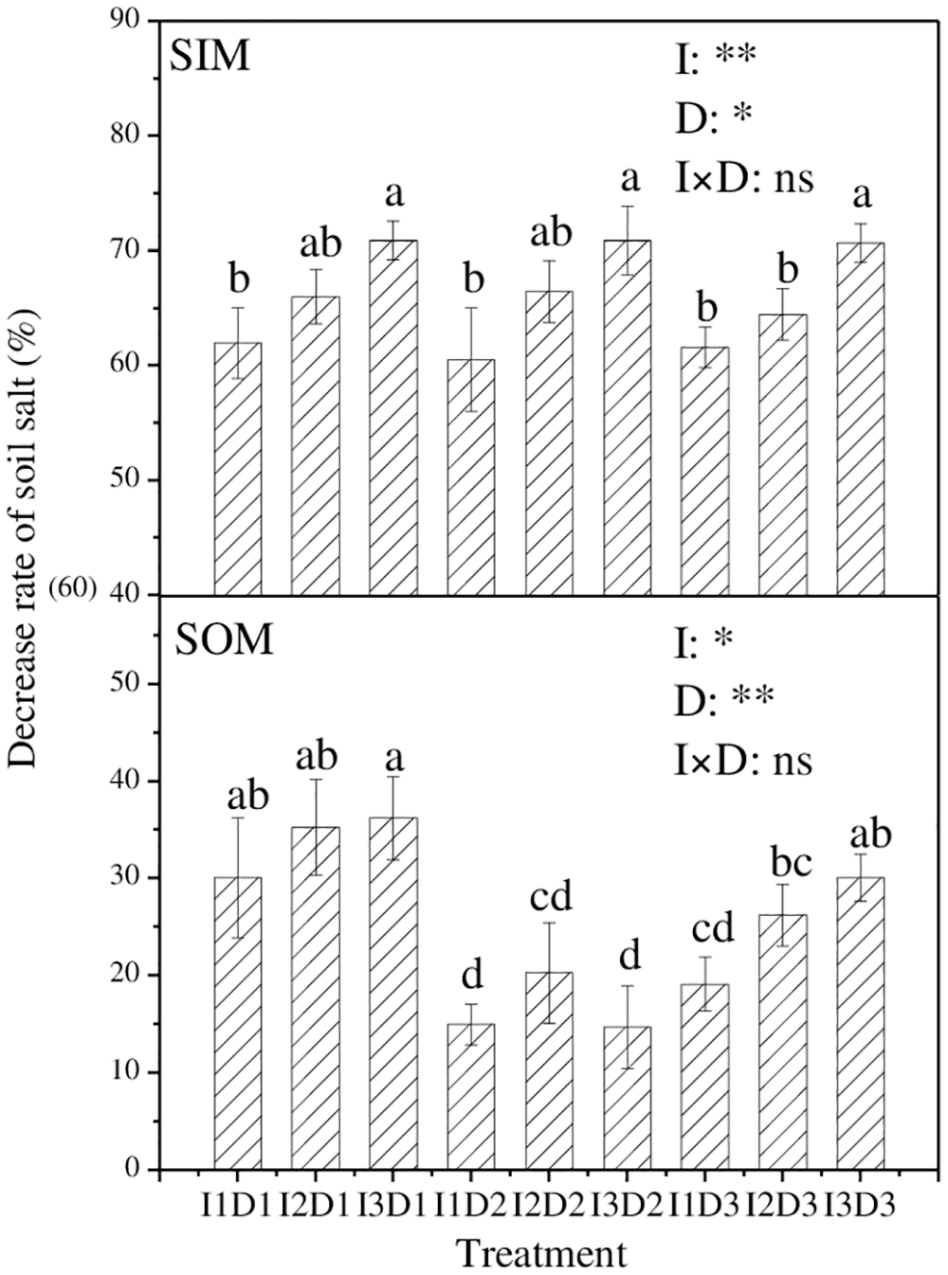
The decreasing rate of soil salt with different treatments. (SIM and SOM represent the sampling location inside and outside the mulch, respectively. The rate was calculated from Jun. 10 to Sept. 21.The values of the decreasing rate are the means of three replications. In the same sampling location (SIM or SOM), the means followed by the same letter (a, b, c) do not differ significantly at the 0.05 level, according to Duncan’s multiple range test. I and D represent quotas of irrigation and depths of drain ditch, respectively. *, **and ns indicate that the experimental treatment has a significant (at 0.05 level) effect, an extremely significant (at 0.01 level) effect and no significant effect on the decreasing rate, respectively.)

Fig 3 shows the decreasing rate of soil salt from 0 to 103 DAT. In SIM, the decreasing rate of soil salt was positively related to the irrigation quota, which was significantly higher under I3 than under I1. The irrigation quota and the ditch depth had an extremely significant (*p≤0*.*01*) effect and a significant (*p≤0*.*05*) effect on the salt-decreasing rate in SIM, respectively, but the combination of the irrigation quota and the ditch depth had no significant effect on it. In SOM, the decreasing rate of soil salt was found to be highest with I3D1 treatment, reaching 36.2%. The irrigation quota and the depth of the drain ditch had a significant (*p≤0*.*05*) effect and an extremely significant (*p≤0*.*01*) effect on the salt-decreasing rate in SOM, respectively, whereas the combination of the irrigation quota and the ditch depth had no significant effect on it. Overall, I3D1 obtained the most satisfactory decreasing rate of soil salt in both SIM and SOM.

### Soil organic matter

**Fig 4:**
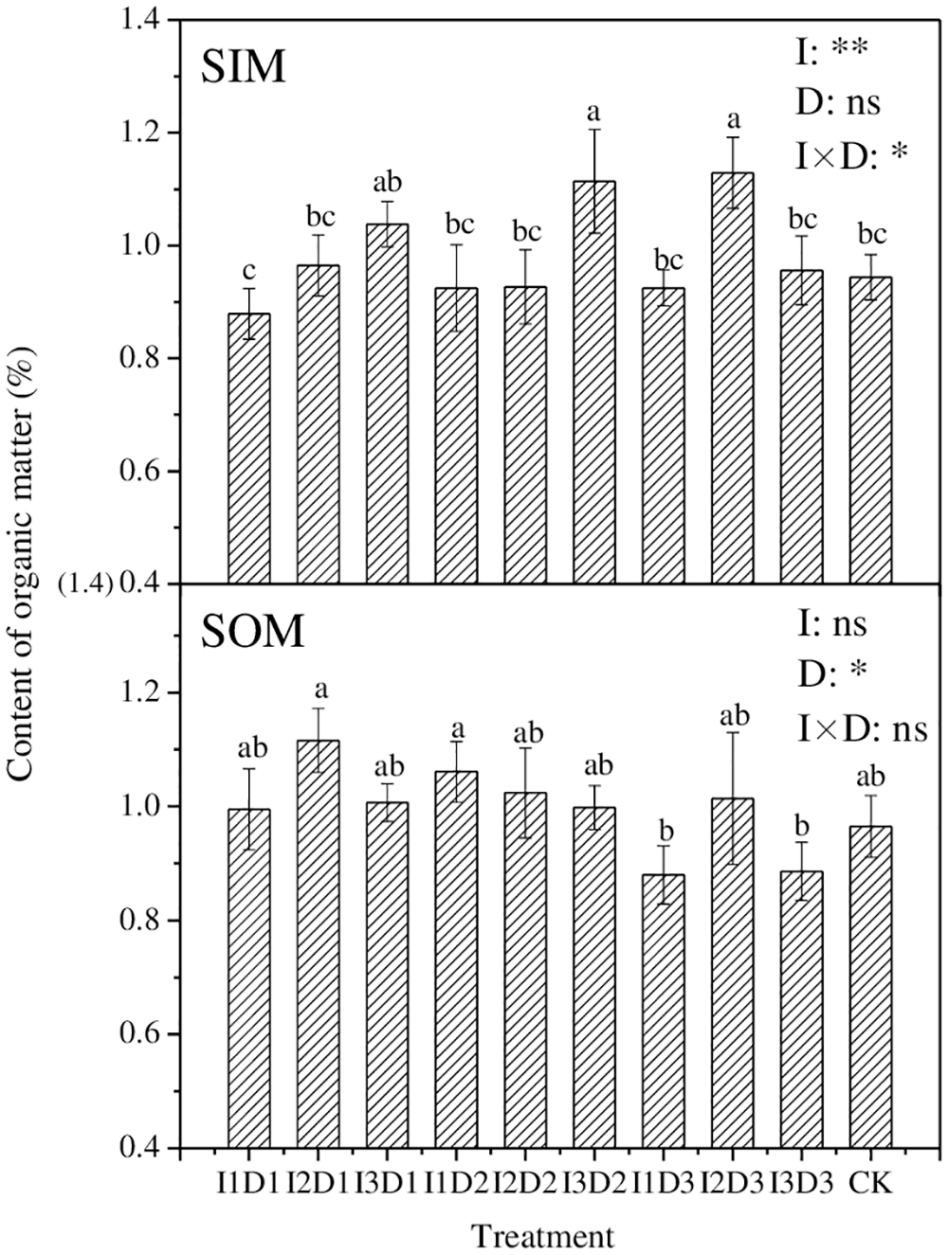
The content of organic matter in soil at a 0-20-cm depth with different treatments. (SIM and SOM represent the sampling location inside and outside the mulch, respectively. Means followed by the same letter (a, b, c) do not differ significantly at the 0.05 level, according to Duncan’s multiple range test. I and D represent quotas of irrigation and depths of drain ditch, respectively. *, **and ns indicate that the experimental treatment has a significant (at 0.05 level) effect, an extremely significant (at 0.01 level) effect, and no significant effect on the organic matter, respectively.)

Fig 4 displays the content of soil organic matter in SIM and SOM for different treatments. In general, the organic matter content in SIM was positively related to the irrigation quota (except for the treatments under D3 ditch depth). I2D3 achieved the greatest organic matter content in SIM and was 19.6% higher than CK. However, the I1D1 treatment, with the lowest irrigation quota and ditch depth, obtained the lowest soil organic matter content—0.879%—in SIM. The organic matter content of soil in SIM was significantly (*p≤0*.*01*) affected by the irrigation quota, as well as significantly (*p≤0*.*05*) affected by the combination of the irrigation quota and the ditch depth.

The contents of soil organic matter in SOM for different treatments ranged from 0.880 to 1.116%, and the greatest value was achieved by I2D1. The organic matter content of soil in SOM was significantly (*p≤0*.*05*) affected by the ditch depth, and it was lower under the D3 depth (I1D3, I2D3 and I3D3) than under the D1 and D2 depths. However, the irrigation quota and the combination of the irrigation quota and the ditch depth had no significant effect on the content of soil organic matter in SOM.

### Soil available nutrients

**Fig 5:**
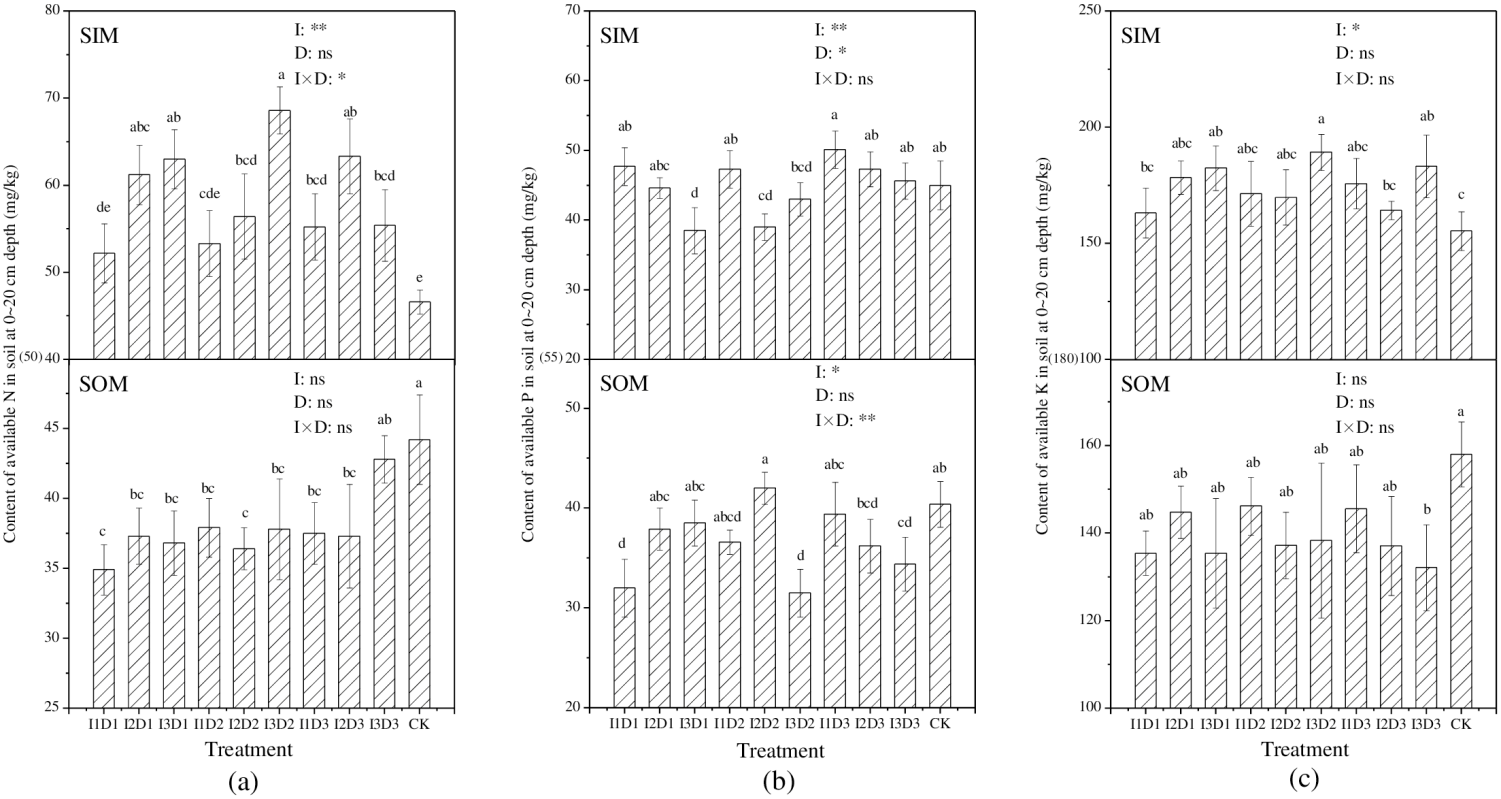
The content of available N (a), P (b) and K (c) in soil at a 0-20-cm depth with different treatments. (SIM and SOM represent the sampling location inside and outside the mulch, respectively. Means followed by the same letter (a, b, c) do not differ significantly at the 0.05 level, according to Duncan’s multiple range test. I and D represent quotas of irrigation and depths of drain ditch, respectively. *, **and ns indicate that the experimental treatment has a significant (at 0.05 level) effect, an extremely significant (at 0.01 level) effect, and no significant effect on the available nutrient, respectively.)

Overall, the content of soil’s available N in SIM increased as the irrigation quota increased (Fig 5). Compared to CK, different treatments increased the content of soil available N in SIM by 12.1–47.1% but decreased it in SOM by 3.4–22.1%. The highest contents of available N in SIM and SOM were achieved by I3D2 and I3D3, respectively—68.6 mg/kg and 42.8 mg/kg. The irrigation quota and the combination of the irrigation quota and the ditch depth had an extremely significant (*p≤0*.*01*) effect and a significant (*p≤0*.*05*) effect on the content of available N in SIM, respectively. However, the irrigation quota, the ditch depth and their combination had no significant effect on the content of available N in SOM.

The contents of soil’s available P in SIM with different treatments were in a range of 38.5 to 50.1 mg/kg. Several treatments decreased the soil’s available P in SIM compared to CK, and these treatments were found under the irrigation quota of I2 or I3, indicating that the higher irrigation was disadvantageous to reserving available P in SIM. For SOM, soil’s available P content with different treatments ranged from 31.5 to 42.0 mg/kg, which were lower than CK (except for I2D2). The irrigation quota had an extremely significant (*p≤0*.*01*) effect and a significant (*p≤0*.*05*) effect on the available P content in SIM and SOM, respectively, demonstrating that the soil’s available P was sensitive to the irrigations. The ditch depth had a significant (*p≤0*.*05*) effect on the available P content in SIM, but it had no significant effect on that in SOM. The combination of irrigation quota and ditch depth had an extremely significant (*p≤0*.*01*) effect on the available P content in SOM, but it had no significant effect on that in SIM.

The variation regularity of available K with different treatments was similar to that of available N. The content of available K in SIM increased by 5.0–21.9%, and that in SOM decreased by 7.5–16.4%, compared to CK. The greatest values of available K in SIM and SOM were obtained by I3D2 and I1D3; they were 189.2 mg/kg and 145.6 mg/kg, respectively. The irrigation quota had a significant (*p≤0*.*05*) effect on the available K content in SIM, but it had no significant effect on that in SOM. The ditch depth and the combination of the irrigation quota and the ditch depth had no significant effect on the available K content in SIM or SOM.

### Performance of tomato plant

Plant height, stem diameter and leaf area index of tomato under different treatments in the seedling stage (10 DAT), flowering stage (30 DAT) and fruit-set stage (60 DAT) were shown in Table 3. Obviously, the irrigation and drainage treatment enhanced the plant height, stem diameter and the leaf area index tomato in various degrees. The plant height was increased by 3.8~11.5, 3.4~18.3 and 9.4~36.3 cm in corresponding to the seedling, flowering and fruit-set stage. The stem diameter was increased by 0.04~0.12, 0.06~0.27 and 0.27~0.58 cm in corresponding to the seedling, flowering and fruit-set stage. The leaf area index was increased by 0.39~1.76, 1.10~2.90 and 2.80~6.86 in corresponding to the seedling, flowering and fruit-set stage. Meanwhile, it could be found from the result that the irrigation quota significantly affected the plant height, stem diameter and leaf area index of tomato during different periods (except the stem diameter at 10 DAT), but the drainage depth had no significant effect on these three indicators. Moreover, soil salinity had significant effects on the plant height, stem diameter and leaf area index of tomato at 30 and 60 DAT. Therefore, influence of irrigation quota on the tomato growth possibly not only because the irrigation itself but also due to the salt-decreasing effects caused by irrigations.

**Table 3:** Plant height, stem diameter and leaf area index of tomato under different treatments.

Treatment	plant height (cm)			stem diameter (cm)			leaf area index		
	10 DAT	30 DAT	60 DAT	10 DAT	30 DAT	60 DAT	10 DAT	30 DAT	60 DAT
I1D1	17.4±1.5bc	40.2±2.18cd	66.4±3.35c	0.45±0.03ab	0.68±0.07bc	1.05±0.06bc	1.95±0.08c	5.12±0.42cd	9.88±0.55c
I2D1	20.6±1.69b	45.8±3.53bc	73.1±4.82bc	0.51±0.05ab	0.77±0.03ab	1.14±0.07b	2.33±0.13b	5.99±0.15ab	11.23±0.28b
I3D1	24.9±2.12a	51.3±4.07ab	90.1±5.11a	0.49±0.04ab	0.82±0.03a	1.29±0.04a	2.98±0.23a	6.68±0.22a	13.38±0.45a
I1D2	17.8±0.98bc	43.4±2.75c	63.2±4.06cd	0.47±0.06ab	0.65±0.03c	0.98±0.02c	1.87±0.09c	5.34±0.25c	8.78±0.33d
I2D2	18.2±1.06bc	42.6±3.35c	74.9±4.73b	0.53±0.07a	0.73±0.04b	1.08±0.06b	2.17±0.11b	5.32±0.23c	9.62±0.33c
I3D2	21.8±1.92ab	55.1±4.95a	87.2±4.95a	0.53±0.08a	0.86±0.05a	1.16±0.07ab	3.24±0.25a	6.23±0.29ab	11.12±0.41b
I1D3	17.2±0.87c	43.2±3.01c	63.2±3.58c	0.49±0.05ab	0.72±0.06b	1.08±0.04b	2.01±0.12c	4.88±0.11d	9.37±0.56cd
I2D3	21.2±1.38ab	48.7±2.63ab	68.9±4.16bc	0.45±0.04ab	0.73±0.06b	1.17±0.03ab	2.34±0.12b	5.42±0.31c	9.52±0.84cd
I3D3	23.5±2.01ab	50.6±3.08ab	89.9±5.32a	0.46±0.05ab	0.85±0.03a	1.22±0.09a	3.02±0.03a	6.01±0.11b	12.84±0.32a
CK	13.4±0.79d	36.8±1.79d	53.8±3.48d	0.41±0.02b	0.54±0.03d	0.71±0.07d	1.48±0.04d	3.78±0.17e	5.98±0.25e
I	*	*	**	ns	*	*	*	*	**
D	---	---	ns	---	---	ns	---	---	ns
Soil salinity	---	*	*	---	*	*	---	*	**

Note: The values of the plant height, stem diameter and leaf area index are the means of 3 replications. In the same column, the means followed by the same letter (a, b, c, d and e) do not differ significantly at the 5% level, according to Duncan’s multiple range test. Each value is the mean ± SD (n = 3). I and D represent quotas of irrigation and depths of drain ditch, respectively.

*, **and ns indicate that the experimental treatment has a significant (at 0.05 level) effect, an extremely significant (at 0.01 level) effect and no significant effect on the indicators, respectively. DAT represents the days after transplanted.

### Tomato yield

**Fig 6:**
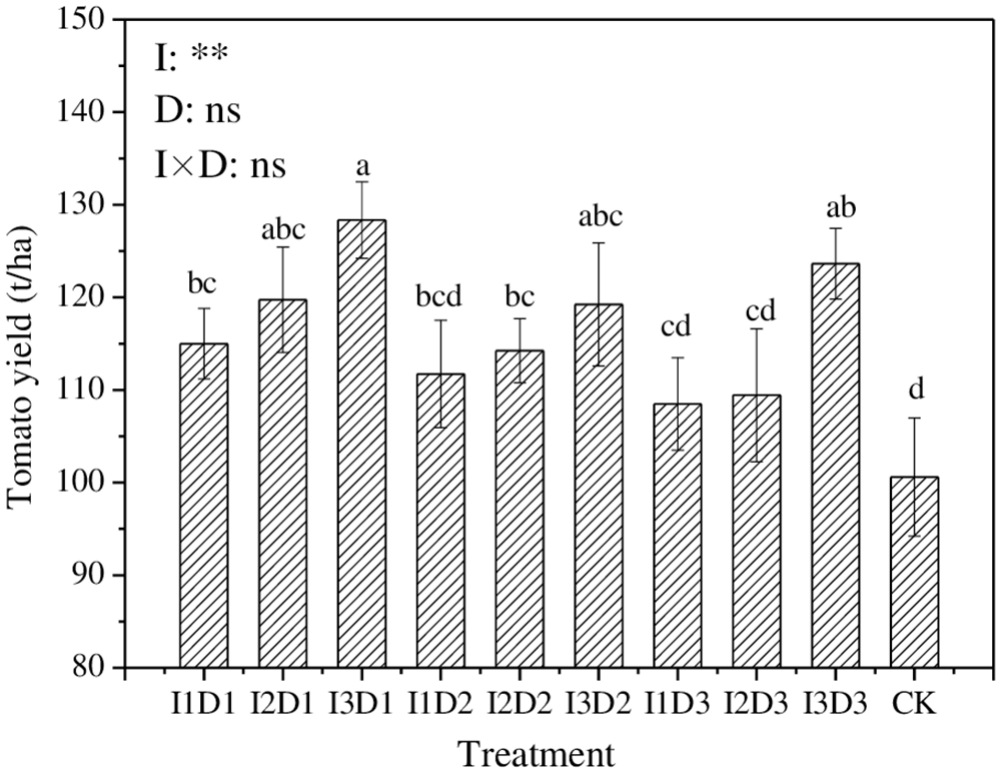
The tomato yield with different treatments. (The values of tomato yield are the means of three replications. Means followed by the same letter (a, b, c) do not differ significantly at the 0.05 level, according to Duncan’s multiple range test. I and D represent quotas of irrigation and depths of drain ditch, respectively. **and ns represent indicate the experimental treatment has an extremely significant (at 0.01 level) effect and no significant effect on the tomato yield, respectively.)

The range of tomato yield (Fig 6) was from 108.5 t/ha to 128.3 t/ha for the treatments. The average was 115.0 t/ha, and the greatest yield was achieved by I3D1 treatment. Compared to CK, different treatments raised the tomato yield by 7.9–27.6%. The tomato yield increased as the irrigation quota increased, and it was found to be significantly affected by the irrigation quota (*p≤0*.*01*). However, the tomato yield was not significantly affected by the ditch depth or the combination of the irrigation quota and the ditch depth.

## Discussion

The conflict of land use between urban expansion and agricultural production is becoming more severe in coastal areas of eastern China, and the utilization of coastal land resources has been a new approach to alleviating the decrease in agricultural land. However, in such areas, the soils contain excessive salt, which is harmful to the growth and development of crops. Therefore, salt-decreasing measures should be implemented in these soils before or during crop planting. Among the existing measures, subsurface drainage is considered an effective method to remove salt from soil; it can also restrain salt resalinization by lowering the groundwater table [25]. However, the cost of this approach is relatively high, particularly in purchasing and installing subsurface pipes [26]; therefore, it is difficult to apply and to popularize this method in some rural areas of China. Bio-organic fertilizer is beneficial in improving saline soils, but it takes a long time to reach the desired effects [27]. Isolation laying and straw mulching can limit the movement of salt from the deeper soil layer to the lower soil layer [7], but these methods cannot radically remove salt from soils. Here, we proposed a method that combines mulched drip irrigation with surface drainage, and we analysed the response of soil and tomatoes to different combination treatments.

In this study, the salinity of soil at a 0-60-cm depth in SIM and SOM presented decreasing and increasing trends, respectively. Similar results were observed by Wang [14] in arid agricultural areas in northern China. These findings demonstrate that soil salt moves horizontally from the inside to the outside of mulch under mulched drip irrigation. After the surface drainage on 102 DAT, the soil salinity in SOM increased under some of the treatments, which is likely because the resalinization effect was more significant than the desalination effect [28]. For SOM, the average decreasing rate of soil salt under a D1 depth of the drain ditch was found to be higher than that under D2 and D3, which is likely because the lower layer of soil usually accumulated more salt due to the evaporation; the drainage water under D1 could wash those salts in the lower soil layer more adequately. Moreover, the decreasing rate of soil salt in SOM was 14.7–36.2%, which was much lower than the 36.7–63.3% rate reported by Zhou [29]. The discrepancy might be due to the fact that Zhou adopted a higher drainage flow (5 L/s) and a longer drainage duration. Because the soil salt was continuously moving, the actual removing rate of soil salt could not be accurately evaluated judging only from the decreasing rate of soil salinity between two time points. Previous research evaluated the salt-removing rate by measuring the mineral content in water after drainage and then calculating the total content of salt that was washed out [14]. According to this method, we evaluated the total salt amount in the water after drainage, estimated the total amount of salt in soil at a 0-1-m depth based on the original bulk density (1.46 g/cm^3^) and 3.47 g/kg, and finally calculated the total removal rate of salt from soil. The total salt-removal rate in this study was 8.7–13.2%, demonstrating that the treatments obtained a satisfactory effect in removing soil salt. However, although our study compared the salt content of SIM and SOM in the soils at 0–60 cm depth, the analysis of the distribution and migration of soil salinity at various depths, and the accumulation of soil salts in different irrigation quota during the irrigation process should be carried out in further studies.

Soil organic matter was the key factor that affected soil fertility levels. Increasing the content of organic matter in soil can generally be regarded as improving the fertility of soils [30]. In our study, the content of soil organic matter in SIM was slightly lower than that in SOM, which may due to the crop consumption. Overall, the content of soil organic in SIM increased as the irrigation quota increased; this finding agreed with Zhang’s [31] results but was different from the findings of Zhen and Liu [32], who reported that irrigation decreased the content of soil organic matter by promoting its transformation and increasing its amount absorbed by crops. The relationship between the irrigation quota and soil’s organic matter in our study can likely be explained by the fact that the higher irrigation quota increased the soil moisture and restrained the activity of aerobic microorganisms, which led to the anaerobic decomposition and produced the reducing agents; this was conductive to reserving the organic matter in the soil.

Generally, in our study, the content of the soil’s available nutrients in SIM was increased, which is likely because the soil environment under mulched drip irrigation were beneficial for the transformation of mineral nutrition [33]. The moisture and temperature in the film might have promoted the release of available nutrient from fertilizers and soils, and the amount of nutrient increment was larger than that absorbed by the tomato plants. However, the soil’s available nutrients in SOM presented an obvious decreasing trend, indicating that surface drainage could cause the loss of available nutrients in soil. This result was similar to the finding of Sims [34]. It should be noted that the content soil’s available nutrients in SOM was not significantly affected by the depth of drain ditch, perhaps because the flows of drainage water were the same across different treatments. The soils were ploughed uniformly before the experiment so that the nutrient distributed evenly in the soil layer. Further studies that similar to ours should noticed that the available nutrient at different period will be closely related to the tomato growth and yield formation, so it was also important to study nutrient at various growth period of tomato.

Salt stress often results in pernicious effects on crops, such as decreased turgor pressure, a lower speed of cell expansion and damage to chloroplasts, thus reducing the growth rate and photosynthesis. These changes ultimately influence the dry-matter accumulation and yield of crops [35]. In this study, a positive relationship between tomato yield and salt-decreasing rate (in SIM) was detected (*r* = 0.825**). The tomato yield increased as the irrigation quota increased, which might also be related to the better salt-decreasing effect of the higher irrigation quota. Our study found that different treatments increased the tomato yield by 7.9–27.6%. Similar results were obtained by Hanson and May [36] under drip systems.

The irrigation influenced the salt content of soil in SOM. The surface drainage also influenced the salt content of soil in SIM. Therefore, the salt-removing effect of the mulched drip irrigation combined with surface drainage was not a simple addition of the two single effects. This study primarily utilized the directional migration of soil salt under mulched drip irrigation to wash the salt by surface drainage where salt largely accumulated. Otherwise, under a superior irrigation quota and drain depth (particularly the irrigation quota), the tomato yield was increased, whereas the tomato CQI was not significantly affected. Therefore, the combination of mulched drip irrigation and surface drainage was recommended in this study as an effective method to improve soil conditions and to increase crop yield in the coastal area of eastern China. While in practical operation, three times of drainage will be a huge task. If the soils are considered to be washed only once, we recommend that the drainage will be better to be conducted after the last harvest and without the film covered, in order to wash out the accumulated soil in the inner edge of film. The results should be interpreted with caution because this experiment was conducted in eastern China, which has a subtropical climate. The humidity, temperature and illumination conditions might be different in other places. Therefore, similar research related to climate factors needs to be conducted in the future.

## Conclusion

After the last drainage (Sept. 20), the greatest decreasing rates in soil salinity were 70.9% in SIM and 36.2% in SOM; both were achieved by I3D1. The total removal rate of salt from soil at a 0-1-m depth was 8.7–13.2% for the treatments. Compared with CK, the treatments increased the available N and K content of the soil in SIM but decreased it in SOM. For tomato plant, the plant height and the stem diameter was increased significantly by the irrigations but was not significantly affected by the drain ditch depth. The leaf area index was increased by 0.39~1.76, 1.10~2.90 and 2.80~6.86 in corresponding to the seedling, flowering and fruit-set stage. Yield increase rates of 7.9–27.6% were found for the treatments compared to the control with similar amount of applied water.
